# 3-(4-Amino-3-methyl-5-sulfanyl­idene-4,5-dihydro-1*H*-1,2,4-triazol-1-yl)-3-(2-fur­yl)-1-phenyl­propan-1-one

**DOI:** 10.1107/S1600536811020988

**Published:** 2011-06-11

**Authors:** Yan Gao, Lu Wang, He-wen Wang

**Affiliations:** a School of Chemical Engineering, University of Science and Technology Liaoning, Anshan 114051, People’s Republic of China; b College of Food Science and Technology, Nanjing Agricultural University, Nanjing 210095, People’s Republic of China; c College of Chemistry and Applied Chemistry, Huanggang Normal University, Huanggang 438000, People’s Republic of China

## Abstract

In the title mol­ecule, C_16_H_16_N_4_O_2_S, the plane of the 1,2,4-triazole ring forms dihedral angles of 77.9 (2) and 30.0 (2)° with the planes of the furyl and phenyl rings, respectively. Weak inter­molecular N—H⋯S and C—H⋯O hydrogen bonds consolidate the crystal packing.

## Related literature

For the crystal structures of related 1,2,4-triazole-5(4*H*)-thione derivates, see: Al-Tamimi *et al.* (2010 ▶); Fun *et al.* (2009 ▶); Tan *et al.* (2010 ▶); Wang *et al.* (2011 ▶).
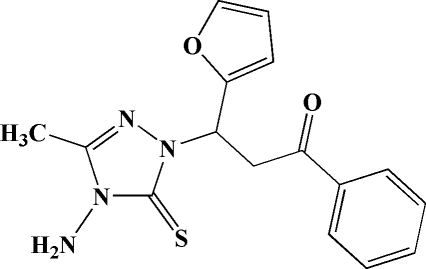


## Experimental

### 

#### Crystal data

C_16_H_16_N_4_O_2_S
*M* *_r_* = 328.39
Monoclinic, 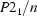

*a* = 7.3702 (8) Å
*b* = 24.131 (2) Å
*c* = 9.240 (1) Å
β = 106.745 (5)°
*V* = 1573.7 (3) Å^3^
*Z* = 4
Mo *K*α radiation
μ = 0.22 mm^−1^
*T* = 113 K
0.20 × 0.18 × 0.12 mm

#### Data collection

Rigaku Saturn CCD area-detector diffractometer
Absorption correction: multi-scan (*CrystalClear*; Rigaku/MSC, 2005 ▶) *T*_min_ = 0.957, *T*_max_ = 0.974
15993 measured reflections
3746 independent reflections
2835 reflections with *I* > 2σ(*I*)
*R*_int_ = 0.053

#### Refinement

*R*[*F* ^2^ > 2σ(*F* ^2^)] = 0.049
*wR*(*F* ^2^) = 0.119
*S* = 1.06
3746 reflections
217 parameters
H atoms treated by a mixture of independent and constrained refinement
Δρ_max_ = 0.37 e Å^−3^
Δρ_min_ = −0.23 e Å^−3^

### 

Data collection: *CrystalClear* (Rigaku/MSC, 2005 ▶); cell refinement: *CrystalClear*; data reduction: *CrystalClear*; program(s) used to solve structure: *SHELXS97* (Sheldrick, 2008 ▶); program(s) used to refine structure: *SHELXL97* (Sheldrick, 2008 ▶); molecular graphics: *SHELXTL* (Sheldrick, 2008 ▶); software used to prepare material for publication: *SHELXTL*.

## Supplementary Material

Crystal structure: contains datablock(s) global, I. DOI: 10.1107/S1600536811020988/cv5087sup1.cif

Structure factors: contains datablock(s) I. DOI: 10.1107/S1600536811020988/cv5087Isup2.hkl

Supplementary material file. DOI: 10.1107/S1600536811020988/cv5087Isup3.cml

Additional supplementary materials:  crystallographic information; 3D view; checkCIF report

## Figures and Tables

**Table 1:** Hydrogen-bond geometry (Å, °)

*D*—H⋯*A*	*D*—H	H⋯*A*	*D*⋯*A*	*D*—H⋯*A*
N4—H4*A*⋯S1^i^	0.95 (3)	2.64 (3)	3.475 (2)	148 (2)
C4—H4*D*⋯O1^ii^	0.99	2.45	3.346 (2)	151
C14—H14⋯O1^iii^	0.95	2.55	3.490 (2)	169

Symmetry codes: (i) 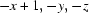; (ii) 
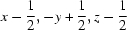
; (iii) 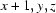.

**Fig. 1.**
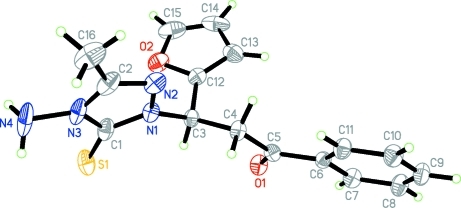
View of (I) showing the atom-labelling scheme. Displacement ellipsoids are drawn at the 55% probability level.

## supplementary crystallographic information

### Comment

In continuation of structural study of 1,2,4-triazole-5(4*H*)-thione derivatives in our group (Wang *et al.*, 2011), we present here the crystal structure of the title compound, (I).

In (I) (Fig. 1), the bond lengths and angles are normal and comparable with those reported for the related 1,2,4-triazole-5(4*H*)- thione derivatives (Al-Tamimi *et al.*, 2010; Fun *et al.*, 2009; Tan *et al.*, 2010; Wang *et al.*, 2011). The 1,2,4-triazole ring makes the dihedral angles of 30.2 (2) and 77.9 (2)° with the phenyl ring and the furanyl ring, respectively. The phenyl  and furanyl rings form a dihedral angle of 71.1 (2) °.

Intermolecular N—H···S hydrogen bond (Table 1) links the adjacent molecule into centrosymmetric dimers, which are further linked by the weak C—H···O interactions (Table 1) into three-dimensional structure.

### Experimental

The title compound was synthesized by the reaction of the 3-(furan-2-yl)-1- phenyl-2-propen-1-one (2.0 mmol) with 4-amino-3-methyl-4*H*-1,2,4-triazole-5- thiol (2.0 mmol) in ethanol. The reaction progress was monitored *via* TLC. The resulting precipitate was filtered off, washed with cold ethanol, dried and purified to give the target product as colorless solid in 84% yield. Crystals of (I) suitable for single-crystal X-ray analysis were grown by slow evaporation of a solution in chloroform-ethanol (1:1).

### Refinement

N-bound H atoms were located in a difference map and isotropically refined. C-bound H atoms were positioned geometrically (C—H = 0.95–0.99 Å) and refined as riding, with *U*_iso_(H) = 1.2-1.5 *U*_eq_(C).

### Crystal data

**Table:**

C_16_H_16_N_4_O_2_S	*F*(000) = 688
*M**_r_* = 328.39	*D*_x_ = 1.386 Mg m^−^^3^
Monoclinic, *P*2_1_/*n*	Mo *K*α radiation, λ = 0.71073 Å
Hall symbol: -P 2yn	Cell parameters from 4962 reflections
*a* = 7.3702 (8) Å	θ = 1.7–27.9°
*b* = 24.131 (2) Å	µ = 0.22 mm^−^^1^
*c* = 9.240 (1) Å	*T* = 113 K
β = 106.745 (5)°	Prism, colorless
*V* = 1573.7 (3) Å^3^	0.20 × 0.18 × 0.12 mm
*Z* = 4	

### Data collection

**Table:**

Rigaku Saturn CCD area-detector diffractometer	3746 independent reflections
Radiation source: rotating anode	2835 reflections with *I* > 2σ(*I*)
multilayer	*R*_int_ = 0.053
Detector resolution: 14.63 pixels mm^-1^	θ_max_ = 27.9°, θ_min_ = 1.7°
φ and ω scans	*h* = −9→9
Absorption correction: multi-scan (*CrystalClear*; Rigaku/MSC, 2005)	*k* = −31→31
*T*_min_ = 0.957, *T*_max_ = 0.974	*l* = −12→10
15993 measured reflections	

### Refinement

**Table:**

Refinement on *F*^2^	Primary atom site location: structure-invariant direct methods
Least-squares matrix: full	Secondary atom site location: difference Fourier map
*R*[*F*^2^ > 2σ(*F*^2^)] = 0.049	Hydrogen site location: inferred from neighbouring sites
*wR*(*F*^2^) = 0.119	H atoms treated by a mixture of independent and constrained refinement
*S* = 1.06	*w* = 1/[σ^2^(*F*_o_^2^) + (0.0574*P*)^2^] where *P* = (*F*_o_^2^ + 2*F*_c_^2^)/3
3746 reflections	(Δ/σ)_max_ < 0.001
217 parameters	Δρ_max_ = 0.37 e Å^−^^3^
0 restraints	Δρ_min_ = −0.23 e Å^−^^3^

### Special details

**Table:**

Geometry. All e.s.d.'s (except the e.s.d. in the dihedral angle between two l.s. planes) are estimated using the full covariance matrix. The cell e.s.d.'s are taken into account individually in the estimation of e.s.d.'s in distances, angles and torsion angles; correlations between e.s.d.'s in cell parameters are only used when they are defined by crystal symmetry. An approximate (isotropic) treatment of cell e.s.d.'s is used for estimating e.s.d.'s involving l.s. planes.
Refinement. Refinement of *F*^2^ against ALL reflections. The weighted *R*-factor *wR* and goodness of fit *S* are based on *F*^2^, conventional *R*-factors *R* are based on *F*, with *F* set to zero for negative *F*^2^. The threshold expression of *F*^2^ > σ(*F*^2^) is used only for calculating *R*-factors(gt) *etc*. and is not relevant to the choice of reflections for refinement. *R*-factors based on *F*^2^ are statistically about twice as large as those based on *F*, and *R*- factors based on ALL data will be even larger.

### Fractional atomic coordinates and isotropic or equivalent isotropic displacement parameters (Å^2^)

**Table:**

	*x*	*y*	*z*	*U*_iso_*/*U*_eq_	
S1	0.32564 (7)	0.044074 (19)	0.12279 (6)	0.03395 (17)	
O1	0.44017 (17)	0.24184 (5)	0.30582 (14)	0.0252 (3)	
O2	0.76075 (17)	0.12393 (5)	0.14479 (15)	0.0302 (3)	
N1	0.3805 (2)	0.13261 (6)	−0.04619 (17)	0.0216 (3)	
N2	0.3656 (2)	0.14722 (6)	−0.19396 (18)	0.0266 (4)	
N3	0.2807 (2)	0.06078 (6)	−0.17722 (18)	0.0298 (4)	
N4	0.2143 (3)	0.00728 (7)	−0.2257 (2)	0.0447 (6)	
H4A	0.312 (4)	−0.0188 (10)	−0.184 (3)	0.065 (8)*	
H4B	0.120 (4)	0.0015 (10)	−0.184 (3)	0.054 (8)*	
C1	0.3301 (2)	0.07965 (7)	−0.0317 (2)	0.0256 (4)	
C2	0.3022 (3)	0.10221 (8)	−0.2710 (2)	0.0304 (5)	
C3	0.4564 (2)	0.17151 (6)	0.0778 (2)	0.0196 (4)	
H3	0.4213	0.1574	0.1679	0.023*	
C4	0.3677 (2)	0.22862 (6)	0.0401 (2)	0.0219 (4)	
H4C	0.4291	0.2481	−0.0277	0.026*	
H4D	0.2315	0.2244	−0.0144	0.026*	
C5	0.3886 (2)	0.26319 (7)	0.1805 (2)	0.0204 (4)	
C6	0.3390 (2)	0.32323 (7)	0.1632 (2)	0.0199 (4)	
C7	0.3636 (3)	0.35495 (7)	0.2932 (2)	0.0255 (4)	
H7	0.4096	0.3380	0.3897	0.031*	
C8	0.3215 (3)	0.41086 (7)	0.2828 (2)	0.0298 (5)	
H8	0.3391	0.4324	0.3718	0.036*	
C9	0.2533 (3)	0.43531 (7)	0.1412 (2)	0.0297 (5)	
H9	0.2260	0.4738	0.1338	0.036*	
C10	0.2250 (3)	0.40402 (7)	0.0111 (2)	0.0289 (4)	
H10	0.1765	0.4209	−0.0852	0.035*	
C11	0.2673 (3)	0.34799 (7)	0.0217 (2)	0.0251 (4)	
H11	0.2475	0.3264	−0.0675	0.030*	
C12	0.6687 (2)	0.17414 (7)	0.1194 (2)	0.0222 (4)	
C13	0.7944 (3)	0.21587 (8)	0.1527 (2)	0.0310 (5)	
H13	0.7673	0.2544	0.1447	0.037*	
C14	0.9781 (3)	0.19038 (10)	0.2031 (2)	0.0388 (5)	
H14	1.0966	0.2089	0.2356	0.047*	
C15	0.9504 (3)	0.13569 (10)	0.1956 (2)	0.0384 (5)	
H15	1.0483	0.1087	0.2219	0.046*	
C16	0.2566 (3)	0.09657 (10)	−0.4376 (2)	0.0435 (6)	
H16A	0.2883	0.1311	−0.4806	0.065*	
H16B	0.1211	0.0889	−0.4801	0.065*	
H16C	0.3303	0.0660	−0.4620	0.065*	

### Atomic displacement parameters (Å^2^)

**Table:**

	*U*^11^	*U*^22^	*U*^33^	*U*^12^	*U*^13^	*U*^23^
S1	0.0401 (3)	0.0202 (2)	0.0366 (3)	−0.00212 (19)	0.0030 (2)	0.0041 (2)
O1	0.0304 (7)	0.0238 (6)	0.0186 (7)	0.0022 (5)	0.0025 (5)	0.0014 (5)
O2	0.0255 (7)	0.0342 (7)	0.0290 (8)	0.0083 (5)	0.0051 (6)	0.0028 (6)
N1	0.0240 (8)	0.0204 (7)	0.0175 (9)	0.0025 (6)	0.0014 (6)	−0.0010 (6)
N2	0.0261 (8)	0.0324 (8)	0.0186 (9)	0.0048 (6)	0.0019 (7)	−0.0012 (7)
N3	0.0307 (9)	0.0222 (7)	0.0295 (10)	0.0064 (6)	−0.0028 (7)	−0.0095 (7)
N4	0.0461 (12)	0.0232 (8)	0.0509 (14)	0.0068 (8)	−0.0081 (11)	−0.0183 (9)
C1	0.0226 (9)	0.0205 (8)	0.0280 (11)	0.0051 (7)	−0.0017 (8)	−0.0039 (8)
C2	0.0243 (10)	0.0376 (10)	0.0247 (11)	0.0106 (8)	−0.0003 (8)	−0.0070 (9)
C3	0.0227 (9)	0.0179 (8)	0.0162 (10)	−0.0002 (7)	0.0027 (7)	−0.0023 (7)
C4	0.0260 (9)	0.0190 (8)	0.0180 (10)	0.0021 (7)	0.0021 (7)	0.0004 (7)
C5	0.0182 (8)	0.0217 (8)	0.0191 (10)	−0.0022 (7)	0.0015 (7)	−0.0008 (7)
C6	0.0184 (8)	0.0192 (8)	0.0212 (10)	−0.0006 (6)	0.0043 (7)	−0.0018 (7)
C7	0.0279 (10)	0.0268 (9)	0.0199 (10)	0.0026 (7)	0.0036 (8)	0.0013 (8)
C8	0.0361 (11)	0.0248 (9)	0.0272 (11)	0.0018 (8)	0.0070 (9)	−0.0055 (8)
C9	0.0374 (11)	0.0202 (9)	0.0320 (12)	0.0033 (8)	0.0106 (9)	−0.0013 (8)
C10	0.0366 (11)	0.0262 (9)	0.0238 (11)	0.0046 (8)	0.0087 (8)	0.0060 (8)
C11	0.0302 (10)	0.0235 (8)	0.0205 (11)	0.0014 (7)	0.0056 (8)	−0.0026 (7)
C12	0.0232 (9)	0.0259 (9)	0.0168 (10)	0.0034 (7)	0.0045 (7)	0.0011 (7)
C13	0.0342 (11)	0.0360 (10)	0.0243 (11)	−0.0091 (8)	0.0105 (9)	−0.0037 (8)
C14	0.0229 (10)	0.0715 (15)	0.0229 (11)	−0.0114 (10)	0.0079 (8)	−0.0037 (11)
C15	0.0207 (10)	0.0649 (15)	0.0277 (12)	0.0103 (10)	0.0038 (8)	0.0039 (11)
C16	0.0357 (12)	0.0648 (14)	0.0250 (13)	0.0099 (10)	0.0008 (9)	−0.0121 (11)

### Geometric parameters (Å, °)

**Table:**

S1—C1	1.674 (2)	C6—C7	1.391 (2)
O1—C5	1.223 (2)	C6—C11	1.396 (2)
O2—C15	1.370 (2)	C7—C8	1.381 (2)
O2—C12	1.3755 (19)	C7—H7	0.9500
N1—C1	1.348 (2)	C8—C9	1.390 (3)
N1—N2	1.383 (2)	C8—H8	0.9500
N1—C3	1.462 (2)	C9—C10	1.383 (3)
N2—C2	1.309 (2)	C9—H9	0.9500
N3—C2	1.362 (3)	C10—C11	1.385 (2)
N3—C1	1.366 (2)	C10—H10	0.9500
N3—N4	1.407 (2)	C11—H11	0.9500
N4—H4A	0.95 (3)	C12—C13	1.343 (2)
N4—H4B	0.89 (2)	C13—C14	1.437 (3)
C2—C16	1.484 (3)	C13—H13	0.9500
C3—C12	1.501 (2)	C14—C15	1.334 (3)
C3—C4	1.522 (2)	C14—H14	0.9500
C3—H3	1.0000	C15—H15	0.9500
C4—C5	1.513 (2)	C16—H16A	0.9800
C4—H4C	0.9900	C16—H16B	0.9800
C4—H4D	0.9900	C16—H16C	0.9800
C5—C6	1.491 (2)		
			
C15—O2—C12	106.30 (14)	C11—C6—C5	122.09 (16)
C1—N1—N2	113.18 (14)	C8—C7—C6	120.44 (17)
C1—N1—C3	125.81 (15)	C8—C7—H7	119.8
N2—N1—C3	120.89 (14)	C6—C7—H7	119.8
C2—N2—N1	103.86 (15)	C7—C8—C9	119.52 (18)
C2—N3—C1	109.54 (15)	C7—C8—H8	120.2
C2—N3—N4	124.36 (17)	C9—C8—H8	120.2
C1—N3—N4	126.09 (18)	C10—C9—C8	120.61 (16)
N3—N4—H4A	109.0 (14)	C10—C9—H9	119.7
N3—N4—H4B	104.4 (16)	C8—C9—H9	119.7
H4A—N4—H4B	108 (2)	C9—C10—C11	119.81 (17)
N1—C1—N3	102.79 (16)	C9—C10—H10	120.1
N1—C1—S1	130.11 (14)	C11—C10—H10	120.1
N3—C1—S1	127.09 (14)	C10—C11—C6	120.04 (17)
N2—C2—N3	110.62 (17)	C10—C11—H11	120.0
N2—C2—C16	125.36 (19)	C6—C11—H11	120.0
N3—C2—C16	124.02 (17)	C13—C12—O2	110.33 (16)
N1—C3—C12	111.29 (14)	C13—C12—C3	133.62 (16)
N1—C3—C4	111.10 (13)	O2—C12—C3	115.67 (14)
C12—C3—C4	111.56 (13)	C12—C13—C14	106.08 (18)
N1—C3—H3	107.6	C12—C13—H13	127.0
C12—C3—H3	107.6	C14—C13—H13	127.0
C4—C3—H3	107.6	C15—C14—C13	106.89 (18)
C5—C4—C3	111.85 (14)	C15—C14—H14	126.6
C5—C4—H4C	109.2	C13—C14—H14	126.6
C3—C4—H4C	109.2	C14—C15—O2	110.40 (17)
C5—C4—H4D	109.2	C14—C15—H15	124.8
C3—C4—H4D	109.2	O2—C15—H15	124.8
H4C—C4—H4D	107.9	C2—C16—H16A	109.5
O1—C5—C6	120.69 (16)	C2—C16—H16B	109.5
O1—C5—C4	120.43 (15)	H16A—C16—H16B	109.5
C6—C5—C4	118.85 (15)	C2—C16—H16C	109.5
C7—C6—C11	119.55 (15)	H16A—C16—H16C	109.5
C7—C6—C5	118.35 (16)	H16B—C16—H16C	109.5
			
C1—N1—N2—C2	0.83 (19)	O1—C5—C6—C7	3.7 (2)
C3—N1—N2—C2	177.05 (14)	C4—C5—C6—C7	−178.26 (15)
N2—N1—C1—N3	−0.38 (19)	O1—C5—C6—C11	−175.51 (16)
C3—N1—C1—N3	−176.37 (15)	C4—C5—C6—C11	2.5 (2)
N2—N1—C1—S1	179.91 (13)	C11—C6—C7—C8	−1.4 (3)
C3—N1—C1—S1	3.9 (3)	C5—C6—C7—C8	179.31 (16)
C2—N3—C1—N1	−0.21 (19)	C6—C7—C8—C9	0.3 (3)
N4—N3—C1—N1	−179.21 (17)	C7—C8—C9—C10	0.9 (3)
C2—N3—C1—S1	179.51 (14)	C8—C9—C10—C11	−0.9 (3)
N4—N3—C1—S1	0.5 (3)	C9—C10—C11—C6	−0.2 (3)
N1—N2—C2—N3	−0.94 (19)	C7—C6—C11—C10	1.4 (3)
N1—N2—C2—C16	178.24 (17)	C5—C6—C11—C10	−179.39 (16)
C1—N3—C2—N2	0.8 (2)	C15—O2—C12—C13	0.0 (2)
N4—N3—C2—N2	179.78 (17)	C15—O2—C12—C3	−173.96 (16)
C1—N3—C2—C16	−178.42 (17)	N1—C3—C12—C13	137.1 (2)
N4—N3—C2—C16	0.6 (3)	C4—C3—C12—C13	12.4 (3)
C1—N1—C3—C12	97.51 (19)	N1—C3—C12—O2	−50.8 (2)
N2—N1—C3—C12	−78.20 (18)	C4—C3—C12—O2	−175.52 (15)
C1—N1—C3—C4	−137.52 (17)	O2—C12—C13—C14	−0.2 (2)
N2—N1—C3—C4	46.8 (2)	C3—C12—C13—C14	172.20 (19)
N1—C3—C4—C5	159.98 (14)	C12—C13—C14—C15	0.4 (2)
C12—C3—C4—C5	−75.20 (19)	C13—C14—C15—O2	−0.4 (2)
C3—C4—C5—O1	−12.1 (2)	C12—O2—C15—C14	0.3 (2)
C3—C4—C5—C6	169.87 (14)		

### Hydrogen-bond geometry (Å, °)

**Table:**

*D*—H···*A*	*D*—H	H···*A*	*D*···*A*	*D*—H···*A*
N4—H4A···S1^i^	0.95 (3)	2.64 (3)	3.475 (2)	148 (2)
C4—H4D···O1^ii^	0.99	2.45	3.346 (2)	151
C14—H14···O1^iii^	0.95	2.55	3.490 (2)	169

Symmetry codes: (i) −*x*+1, −*y*, −*z*; (ii) *x*−1/2, −*y*+1/2, *z*−1/2; (iii) *x*+1, *y*, *z*.
